# Abnormalities in brain structure and behavior in GSK-3alpha mutant mice

**DOI:** 10.1186/1756-6606-2-35

**Published:** 2009-11-19

**Authors:** Oksana Kaidanovich-Beilin, Tatiana V Lipina, Keizo Takao, Matthijs van Eede, Satoko Hattori, Christine Laliberté, Mustafa Khan, Kenichi Okamoto, John W Chambers, Paul J Fletcher, Katrina MacAulay, Bradley W Doble, Mark Henkelman, Tsuyoshi Miyakawa, John Roder, James R Woodgett

**Affiliations:** 1 Samuel Lunenfeld Research Institute, Mount Sinai Hospital, Toronto, ON M5G 1X5, Canada; 2 Division of Systems Medical Science, Institute for Comprehensive Medical Science, Fujita Health University, 1-98 Dengakugakubo, Kutsukake-cho, Toyoake 470-1192, Japan; 3 Genetic Engineering and Functional Genomics Group, Frontier Technology Center, Graduate School of Medicine, Kyoto University, Yoshida-Konoe-cho, Sakyo-ku, Kyoto 606-8501, Japan; 4 Japan Science and Technology Agency (JST), Core Research for Evolutional Science and Technology (CREST), Kawaguchi, Japan; 5 Section of Behavior Analysis, Center for Genetic Analysis of Behavior, National Institute for Physiological Sciences, Okazaki, Japan; 6 Mouse Imaging Center, Hospital for Sick Children, Toronto, ON M5G 1X8, Canada; 7 Center for Addiction and Mental Health, Toronto, Canada; 8 McMaster Stem Cell and Cancer Research Institute, McMaster University, Hamilton, ON L8N 3Z5, Canada; 9 University of Toronto, Departments of Medical Biophysics, Psychology, Psychiatry and Molecular & Medical Genetics, Toronto, ON M5S 1A1, Canada

## Abstract

**Background:**

Glycogen synthase kinase-3 (GSK-3) is a widely expressed and highly conserved serine/threonine protein kinase encoded by two genes that generate two related proteins: GSK-3α and GSK-3β. Mice lacking a functional GSK-3α gene were engineered in our laboratory; they are viable and display insulin sensitivity. In this study, we have characterized brain functions of GSK-3α KO mice by using a well-established battery of behavioral tests together with neurochemical and neuroanatomical analysis.

**Results:**

Similar to the previously described behaviours of GSK-3β^+/-^mice, GSK-3α mutants display decreased exploratory activity, decreased immobility time and reduced aggressive behavior. However, genetic inactivation of the GSK-3α gene was associated with: decreased locomotion and impaired motor coordination, increased grooming activity, loss of social motivation and novelty; enhanced sensorimotor gating and impaired associated memory and coordination. GSK-3α KO mice exhibited a deficit in fear conditioning, however memory formation as assessed by a passive avoidance test was normal, suggesting that the animals are sensitized for active avoidance of a highly aversive stimulus in the fear-conditioning paradigm. Changes in cerebellar structure and function were observed in mutant mice along with a significant decrease of the number and size of Purkinje cells.

**Conclusion:**

Taken together, these data support a role for the GSK-3α gene in CNS functioning and possible involvement in the development of psychiatric disorders.

## Background

Glycogen synthase kinase-3 is an evolutionary conserved, ubiquitous serine/threonine protein kinase, belonging to the CMCG family of the proline-directed kinases (Cyclin-dependent kinases (CDKs), Mitogen-activated protein kinases (MAPKs), Glycogen synthase kinases (GSKs), and CDK-like kinases (CLKs)). The enzyme was first isolated and purified as an activity capable of phosphorylating and inhibiting glycogen synthase [1,2]. Two main GSK-3 isoenzymes exist - α and β, which differ in their N- and C-terminal regions, while being highly homologous within their kinase domains [3]. The major structural difference between the two is an amino-terminal, glycine-rich extension that is present in GSK-3α and the two gene products share only 36% identity in their last 76 C-terminal residues [3]. In mammals, both isoforms are ubiquitously expressed at the RNA and protein level. GSK-3 is abundant in the brain, both in neurons and glia [4]. Both kinases have similar substrate specificities, regulating a number of transcription factors, regulatory enzymes, and structural proteins [5]. The mechanisms of GSK-3 regulation are not fully understood; precise control appears to be achieved by a combination of phosphorylation, localization, and interactions with GSK-3-binding proteins [6]. Unlike classical protein kinases, GSK-3 is active under resting conditions and is primarily regulated by inactivation. In recent years numerous studies have indicated that GSK-3 acts downstream to suppress the activity of several prominent pathways such as Wnt signaling, PI-3 kinase and neurotrophic pathways [5,7,8]. GSK-3 and some of its substrates (MAP1B, Tau, presenilin-1, CREB, β-catenin) [9-14] have been implicated in fundamental brain functions, such as neurogenesis, development of neuronal tissue, regulation of synaptogenesis and axonal growth cone collapse [11,15-17], cytoskeletal stabilization [18-21], cell adhesion [22], energy metabolism [23], synaptic plasticity and memory formation [24-28], as well as neurotransmitter signaling [29-31] and circadian rhythms [32,33]. Dysregulation of GSK-3-substrate-mediated phosphorylation and associated signaling pathways have been implicated in the pathogenesis of psychiatric and neurodegenerative diseases, such as schizophrenia, Alzheimer's disease, bipolar mood disorder and ADHD [34-38]. Lithium, the first clinically utilized drug identified to inhibit GSK-3 in a selective manner [39,40] is widely used to augment antipsychotic treatment in patients with schizophrenia [41,42], Alzheimer's Disease [43], Amyotrophic Lateral Sclerosis [44]; and bipolar mood disorders [45-47].

Several genetic mouse models have been generated to study the role of GSK-3 isoenzymes. Mice mutant for GSK-3β die *in utero*, indicating a critical role of GSK-3β in embryogenesis as well as illustrating the non-equivalent roles of the two genes [48,49]. GSK-3β heterozygous mice are viable, morphologically normal and have been tested extensively. These mice have been shown to exhibit a lithium-mimetic antidepressant-like state [29,50], reduced exploratory activity [50] with no changes in total activity, but reduced responsiveness to amphetamine [29,50], no change in sensorimotor function [50,51], increased anxiety [51], and reduced aggressive behavior [52]; as well as normal coordination and balance [50,51]. Kimura *et al*. [53] recently showed that GSK-3β^+/- ^mice have retrograde amnesia and impaired memory reconsolidation. Overexpression of GSK-3β reproduces behavioral correlates of hyperactivity and mania [54]. Selective deletion of both GSK-3α and β in the developing nervous system (via nestin-Cre mediated excision) results in dramatic hyperproliferation of neuronal progenitors along the entire rostocaudal extent of the neuraxis; suppressed neurogenesis, dysregulation of β-catenin and Notch signaling, and disruption of polarity [55]. GSK-α S21A, β S9A knock-in mice have impaired neurogenesis due to a reported deficiency in external support for neural precursor cells [56]. Mice lacking both alleles of GSK-3α are viable and have improved glucose tolerance, elevated hepatic glycogen storage and insulin sensitivity [57].

In the present study, we have analyzed the neuronal function of GSK-3α KO mice at anatomical, histological, biochemical and behavioral levels. To assess the possible utility of these animals in modeling psychiatric disorders, the animals were subjected to a comprehensive battery of behavioral tests. GSK-3α KO mice were evaluated for novelty-induced locomotor activity, responses to open field situations, stress in the elevated plus maze (EPM) and light/dark transition test, social affiliation/novelty, forced swim and tail suspension tests, sensorimotor gating ability by prepulse inhibition of acoustic startle response (PPI/ASR), attention-related processes in the latent inhibition (LI) and learning/memory in the fear conditioning (FC) and passive avoidance paradigms. We found several behavioral anomalies in GSK-3α^-/- ^animals which were similar to the previously characterized GSK-3β^+/- ^mice. However increased sensitivity to aversive cues, decreased sociability, impaired associative memory and abnormal cerebellar functions were additionally and specifically observed in GSK-3α^-/- ^mutants along with brain physiological differences. These findings reveal for the first time the potential role of GSK-3α in CNS function and pathogenesis of psychiatric disorders.

## Methods

### Animals and experimental design

Except where indicated, animals were housed at the Toronto Centre for Phenogenomics (TCP). GSK-3α null (KO) mutants and their wild-type (WT) littermates, were generated as previously described [57]. GSK-3α KO mice were 6 generations backcrossed to C57BL/6 mice. GSK-3α mice were mated heterozygously and at weaning, littermates of mixed genotypes were housed by gender in groups of 3 to 5 per cage under a 12 hour light/dark cycle (lights on at 07.00) with *ad libitum *food (Purina mouse chow) and water. GSK-3α male and female mice were tested at 2-6 months of age. Behavioral testing was conducted between 09.00 and 18.00 hours. Experimenters were blind to genotype, which was determined after data collection. Between subjects and after all tests, all apparatus was cleaned with Clidox and 70% ethanol to prevent bias due to olfactory cues. We used 6 independent groups of KO and WT littermate control mice for behavioral tests. All behavioral testing procedures were initiated from less-stressful to more aversive, where each test was separated from each other by 2 days at least. Prior to all experiments, mice were left undisturbed in the room for 30 min to allow acclimation. The sequence of experiments for the first three sets of animals was: elevated plus maze (EPM), open field (OF), social interaction, startle response/prepulse inhibition (PPI), forced swim test (FST). The fourth set comprised: olfactory bulb test, resident intruder (RI). The fifth group: tail-flick, latent inhibition (LI). Sixth group: olfactory bulb test, tail suspension test (TST), fear conditioning (FC). In addition, six independent sets of naïve mice were used once for separate procedures: 1) MRI; 2) HPLC; 3) Basal and stress-induced corticosteroid measurements; 4) histological studies (Calbindin staining); 5) rotarod; 6) light/dark transition test, passive avoidance experiments. The latter tests were performed in Center for Genetic Analysis of Behavior, National Institute for Physiological Sciences, Okazaki, Japan using mice shipped from Toronto. In Okazaki (NIPS), only male mice that were single housed were used. Animals were 8-9 month old when tests were conducted. Data collection for most behavioral tests (except where noted) was performed by OBSERVER 5.0 software (Noldus Information Technology, Wageningen, the Netherlands). All procedures involving animals were approved by the Animal Care and Use Committee of TCP and were conducted in accordance with the requirements of the Province of Ontario Animals for Research Act 1971 and the Canadian Council on Animal Care. The animal research protocol used in Okazaki (NIPS) was approved by the Institutional Animal Care and Use committee of National Institute for Physiological Sciences.

### Behavioral tests

#### Elevated plus maze (EPM)

Experiments were conducted in a dimly lit room with a light intensity on the central platform of 210 lux [58]. During a 5-min observation period, the number of entries (defined as four paws into an arm) and the amount of time spent in open arm, closed arm and the central platform were scored by the observer. The total number of entries for each subject was collected. These data are presented as percentage time spent in closed or open arm/total duration of experiment ×100.

### Light/dark transition test

This test was conducted as previously described [59,60]. The apparatus consisted of a cage (21 cm × 42 cm × 25 cm) divided into two sections of equal size by a partition containing a door (O'Hara & Co., Tokyo, Japan). One chamber was brightly illuminated (390 lux), whereas the other chamber was dark (2 lux). Mice were placed into the dark side and allowed to move freely between the two chambers with the door open for 10 min. The total number of transitions between chambers, time spent in each side, first latency to enter the light side and distance traveled were recorded automatically.

### New environment test

The activity cage consisted of a cubical box (Versa Max system) with a floor equipped with horizontal and vertical infrared sensors. Each mouse was placed individually into the center of the activity cage for 5 min (as described in [61]) and the following behavioral measures recorded: (1) latency (seconds) to first escape from the center; (2) length of time of immobility periods (seconds); (3) time spent self grooming (seconds); (4) number of risk assessment behaviors involving the mouse stretching its body from corners/wall toward the center; (5) horizontal and (6) vertical activity. Exploratory activity and walking were recorded separately for the central and peripheral field of the open arena and the ratio between central and peripheral activity was calculated.

### Open field test

The activity cage was similar to that used for the new environment test with a floor equipped with horizontal and vertical infrared sensors. The chamber of the test was illuminated at 100 lux. Each mouse was placed individually into the center of the activity cage and motor activity measured by counting the number of horizontal and vertical beam breaks during the 30 min testing period.

### Prepulse inhibition (PPI) of acoustic startle response

PPI testing was conducted in four foam-lined (sound damping) isolation chambers (Med. Associates Inc., Startle Reflex System, St Albans, VT). All events were recorded and controlled by Med Associates software (Startle Reflex package). During the test, the animal was confined to the holder. Background noise was set at 65 dB. Five types of trials were used. Pulse alone trials (P) consisted of a single white noise burst (120 dB, 40 ms). The prepulse + pulse trials (PP69P, PP73P, PP81P) consisting of a prepulse of noise (20 ms at 69, 73, or 81 dB, respectively) followed 100 ms after prepulse onset by a startling pulse (120 dB, 40 ms). No-stimulus (NS) trials consisted of background noise only. Sessions were structured as follows: (1) 15-min acclimation at background noise level; (2) five P trials; (3) 10 blocks each containing all five trials (P, PP69P, PP73P, PP81P, NS) in pseudorandom order; and (4) five P trials. Intertrials intervals were distributed between 12 and 30 s. The force intensity for each trial was recorded as the startle level. The percentage PPI induced by each prepulse intensity was calculated as [1-(startle amplitude on prepulse trial)/(startle amplitude on pulse alone)] × 100%. Startle amplitude in this formula was calculated as the average response to all of the pulse alone trials, excluding the first and last blocks of five pulse alone trials.

### Latent inhibition (LI)

The LI procedure was used as previously described [62]. All events were programmed by the MED-PC software. Before the beginning of each LI experiment, mice were weighed and water was removed from the cages for 24 h. They were then trained to drink in the experimental chamber for 5 days, 15 min per day (training period). Body weights were monitored daily throughout all behavioral testing and maintained at no lower than 80% of the initial body weight. For each daily training session, mice were acclimated to the chamber without access to the sipper tube for 5 min then the guillotine door was opened. Latency to the first lick and number of licks were recorded for 15 min. The LI procedure was conducted on days 6-9 and consisted of the following stages:

#### Pre-exposure

The PE (pre-exposure) mice received 40 white noise presentations with an interstimulus interval of 60 s. The NPE (non pre-exposure) mice were confined to the chamber for an identical period of time without receiving the stimuli.

#### Conditioning

All mice received conditioning to the noise stimulus. To estimate baseline performance 2 noise-shock pairings were used and 4 noise-shock pairings were used to disrupt LI in order to estimate ability of gene to facilitate LI. Five minutes after the start of the session, a 10-s white noise was followed by a 1-s 0.37 mA foot shock. The noise-shock pairings were given 5 min apart. After the last pairing, mice were left in the experimental chamber for an additional 5 min. Mice received 15 min access to water in their home cages after pre-exposure and conditioning sessions.

#### Lick retraining

Mice were given a 15-min drinking session as during the training period. Data from mice that failed to complete 100 licks were dropped from the analysis.

#### Test

Each mouse was placed in the chamber with access to the sipper tube. When the mouse completed 75 licks the noise was presented and lasted until the mouse reached lick 101. The following times were recorded: Time to first lick, time to complete licks 50-75 (before noise onset; A period), and time to complete licks 76-101 (after noise onset; B period). Degree of lick suppression was calculated as a suppression ratio A/(A + B). A lower suppression score indicates a stronger suppression of drinking. LI consists of lower suppression of drinking (higher suppression ratio) in the pre-exposed compared to the non-pre-exposed mice.

### Contextual and cued Pavlovian fear conditioning (FC)

Fear-conditioning evaluation was performed in a sound-attenuated chamber (Med Associates, St. Albans, VT) equipped with a computer-controlled fear conditioning system (Actimetrics, Wilmette, IL). Freezing behavior, defined as the complete absence of any movement except for respiration and heartbeat, was measured during the context and cued conditioning tests at 0.25 second intervals by using FreezeFrame automated fear conditioning software (ACTIMETRICS software, FREEZEFRAME v. 1.6e). Tests subjects were removed from their home cage and allowed to explore for 2 min. Conditioning consisted of a single pairing of an auditory cue (3600 Hz, 80 dB) with a foot shock (1 mV scrambled). The auditory cue was present 2 min after the training session started and was 30 seconds in duration. The foot shock was delivered continuously during the last 2 seconds of the auditory cue. The subject was removed from the chamber 30 seconds later and returned to its home cage. Approximately 24 h later, each subject was returned to the test chamber and monitored for 5 min. Two hours later, the context was altered and each subject was placed into the altered chamber and allowed 3 min for exploration, after which the auditory tone cue of 3 min was delivered.

### Passive avoidance test

Passive avoidance test was assessed in a two-compartment box with a shock grid (O'Hara & Co., Tokyo, Japan) [63]. This task allows measurement of avoidance behavior and learning abilities in mice. The box consisted of a bright compartment and a dark compartment, separated by a guillotine door. After 30 min adaptation to an experimental room, the subject was placed in the light part, and latency to enter the dark compartment was measured. On entry into the dark compartment, the door was closed and a 3 s footshock (0.3 mA) was applied. The mouse was then removed from the box and placed in its home cage. Twenty-four hours later, the mouse was again placed in the light box and latency to enter the dark compartment was measured. The retention test was performed 35 days after second test.

### Forced swim test

The protocol was performed as described by Cryan *et al*. [64]. The mice were released individually into a transparent plastic cylinder (25 cm height, 18 cm diameter), which contained water at 25°C to the depth of 18 cm. The experiment lasted 6 min, and an observer scored the following parameters in the last 4 min of the trial: (1) active swimming (including crossing the quadrants of the container) and (2) floating (no limb movement and making only minimal movements to keep the head above the water). Each mouse was allowed to dry after the test, and the water was changed between subjects.

### Tail suspension test

This procedure was followed as previously described by Steru *et al*. [65]. Scotch tape pasted to the tip of the tail (~1 cm) was used to securely fasten the mice to a flat wooden surface located 50 cm above the ground. Active hanging and immobility, defined by presence or absence of limb movement, were recorded over a 6 min period.

### Accelerating rotarod

For this experiment, an Economex Rotarod apparatus was used (Columbus Instruments, Columbus, OH, USA). The original 3 cm ribbed plastic rotating axle was divided into four adjustable flanges, which enabled testing a maximum of four mice simultaneously. The rod is suspended at a height of 30 cm above the plastic surface. Mice are placed on top of the rod, facing away from the experimenter. In this orientation, forward locomotion opposite to rotation of the rod is necessary to avoid falling. During the stationary mode, each mouse was first observed for 10 seconds without any rotation. The axle was then adjusted for a constant motor speed of 5 r.p.m., and each mouse observed for a total of 10 seconds (fixed speed mode). Next, beginning at 5 r.p.m., the rotation gradually increased in increments of 0.1 r.p.m. per second and the latency to fall off the axle was recorded in seconds for each mouse for the maximum period of 300 seconds (accelerating speed mode). The mean latency was then calculated by averaging the latency for three consecutive trials [66]. The stationary and fixed speed mode sessions were training periods and allowed the animals to become accustomed to the apparatus. Impaired performance in these sessions served as early indicators of abnormality. The accelerated Rotarod procedure was repeated for 3 constitutive days by 3 trials per day to measure motor learning.

### Crawley's sociability and preference for social novelty test

Two identical transparent Perspex cylinders (each 8 cm diameter, 13 cm tall) with removable, black Perspex lids stood vertically inside the apparatus, one in each side chamber. Each cylinder was perforated with multiple holes (1 cm diameter) to allow for air exchange between the interior and exterior of the cylinder. Briefly, the subject mouse was first placed at the center of the middle chamber. After a 5 min adaptation period in which the subject was free to explore each chamber, an unfamiliar mouse (male C57BL/6J, "stranger 1") was placed inside a cylinder in one of the side chambers. Containing the stranger mouse in the cylinder ensures that social approaches were initiated by the subject mouse and was investigatory only, without direct physical contact. Time spent by the test mouse in each side chamber was recorded over a 10-min period, to estimate social novelty and motivation (session I). Another unfamiliar mouse ("stranger 2") was then placed inside an identical cylinder in the opposite side chamber, and the activity of the test mouse was likewise recorded for a further 10 min, to evaluate social memory (session II). The test mouse was considered to be in the chamber when its head and two front paws entered the chamber.

### Resident-intruder test

Aggression was assessed using the resident-intruder test in isolated male mice, essentially as previously described [67]. Males were housed individually for 4 weeks before assessment, which was performed over three sessions in the same day. Intruders (unfamiliar socially housed C57BL/6J male mice, age and weight matched with each resident) were individually placed in the resident home cage for a 10-min test session and observation was started. The latency, duration and number of events were recorded as: aggressive behavior (contact between the resident and the intruder such as biting or wrestling and aggressive grooming of partner) or social interest behavior (following and sniffing of partner). A different intruder animal was used for each resident. Animals that did not attack the intruder were given an attack latency of 10 min.

### Measurement of brain monoamines and their metabolites

Levels of catecholamine neurotransmitters and their metabolites, including dopamine (DA), 4-hydroxy-3- methoxyphenylacetic acid (HVA), norepinephrine (NE), normetanephrine (NM), serotonin (5-HT) and 5-hydroxyindoleacetic acid (5-HIAA), were quantified by HPLC with electrochemical detection. Analyses were performed on a system consisting of a Thermo Separation Products (TSP) P4000 pump, a TSP AS3000 autosampler with cooling unit, an ESA Coulochem II electrochemical detector (ESA 5011A Analytical Cell and 5020 Guard Cell) and a Spectra Physics 4290 Integrator connected to a PC running TSP WOW chromatography software. The mobile phase, an aqueous mixture of 0.098 M glacial acetic acid, 0.09 M sodium acetate (pH 3.7), 0.118 mM EDTA, 8% methanol and 0.8 mM octane sulphonate was delivered at a flow rate of 0.8 ml/min. Separation of the 100 μl samples was performed on an ACE 250 × 4.6 mm column with Ace C18, 5 μm stationary phase. Peak heights recorded at E2 were detected using electrode potentials as follows (Guard cell +450 mV: Analytical cell E1+100 mV; E2 -400 mV). Quantification of monoamines was performed on 0.1 N perchloric acid extracts in a procedure involving two 30 min. runs per sample. An appropriately diluted sample was run followed by a second run consisting of 1/2 sample and 1/2 standard cocktail (pure monoamines and metabolites in concentrations of 10 pg/μl). Monoamine and metabolite levels in ng/mg tissue wet weight were then calculated.

### Magnetic resonance imaging

#### Mice and brain sample preparation

GSK-3α KO mice (n = 9) and their WT litter mates (n = 9) were anesthetized at 9-10 weeks of age with a combination of Ketamine (Pfizer, Kirkland, QC) (100 mg/kg) and Rompun (Bayer Inc., Toronto, ON) (20 mg/kg) via intraperitoneal injection. One wild type brain sample was excluded from the study due to sample preparation artifacts. A previously described sample preparation protocol for scanning was used [68]. Thoracic cavities were exposed, and the animals were perfused through the left ventricle with 30 ml of phosphate-buffered saline (PBS) (pH 7.4) containing 2 mM ProHance^® ^(gadoteridol, Bracco Diagnostics Inc., Princeton, NJ) contrast agent solution at room temperature (25°C) at a rate of approximately 1.0 ml/min. This was followed by infusion with 30 ml of iced 4% paraformaldehyde (PFA) in PBS containing 2 mM ProHance^® ^at the same rate. Following perfusion, the heads were removed along with the skin, lower jaw, ears and the cartilaginous nose tip. The remaining skull structures were allowed to postfix in 4% PFA containing 2 mM ProHance^® ^at 4°C for 12 h. The skulls were transferred to a PBS and 0.02% sodium azide and 2 mM ProHance^® ^solution at 4°C on a rotator platform until they were scanned.

#### Imaging

A multi-channel 7.0 Tesla MRI scanner (Varian Inc., Palo Alto, CA) [69] with a 6 cm inner bore diameter insert gradient set was used to acquire anatomical images of brains within skulls. Prior to imaging, the samples were removed from the contrast agent solution, blotted and placed into 13 mm diameter plastic tubes filled with a proton-free susceptibility-matching fluid (Fluorinert FC-77, 3 M Corp., St. Paul, MN). Three custom-built, 14 mm diameter solenoid coils with a length of 18.3 mm and over wound ends were used to image three brains in parallel. Parameters used in the scans were optimized for image efficiency and grey/white matter contrast: a T2-weighted, 3D fast spin-echo sequence, with TR/TE = 325/32 ms, four averages, field-of-view 14 × 14 × 25 mm and matrix size = 432 × 432 × 780 giving an image with 32 μm isotropic voxels. Total imaging time was 11.3 hours.

#### Image Processing

The 32 μm isotropic resolution T2-weighted MRI scans were non-linearly aligned to a 3D atlas of the mouse brain with 62 structures identified [70]. This process consisted of an initial step in which all of the MRI scans were non-linearly aligned to each other using an unbiased groupwise registration algorithm [71]. Briefly, rigid body registration was carried out towards a preexisting image based on the same mouse strain. All possible pair-wise 12-parameter registrations were then carried out to create an unbiased linear average model of the entire data set. All images were subsequently non-linearly aligned towards the 12-parameter average. The resulting registered MRIs were resampled and averaged [71,72]. This iterative procedure was repeated for an additional five generations with ever finer deformation grid-point spacing. The end-result is to have all 18 scans deformed into exact alignment with each other in an unbiased fashion. This allows for the analysis of the deformations needed to take each mouse's anatomy into this final atlas space, the goal being to model how the deformation fields relate to genotype. Correspondence with the 3D atlas was obtained by non-linear alignment of the final stage average MRI with the 40-mouse average MRI upon which the atlas is based [70].

#### Analysis

Local differences in brain shape related to genotype were assessed by analysis of the deformation fields [73,74]. To reduce random noise and assure normality under the central limit theorem, the transformation data was blurred prior to analysis with a Gaussian kernel with a full width at half maximum of 1 mm, and the logarithm of the Jacobian was computed for univariate statistical comparison at every image point. This statistical analysis results in millions of separate statistical tests. In order to account for an inflated type I error, the False Discovery Rate (FDR) technique was applied [75] with a 10% FDR threshold. The threshold corresponded to an uncorrected *P*-value of 0.0024. The interpretation of these results is that, on average, 10% of the voxels shown as significant will be false positives. The volume for each anatomical structure defined in the atlas was computed for each mouse by integrating the Jacobian of the transformation mapping the atlas image to the image for that mouse. This procedure has previously been shown to provide volume estimates comparable to those obtained by standard stereological methods using tissue sections [76].

### Immunohistochemistry

To detect Purkinje cells, Calbindin immunohistochemistry was performed on sections of the cerebellum. Eight-week old KO and their WT littermate controls (n = 3 of each) were sacrificed and brains were removed and putt into 4% PFA. Paraffin-embedded coronal sections of cerebellum were deparaffinized with xylene, rehydrated in graduated ethanols, and then washed in phosphate-buffered saline. After citrate buffer antigen retrieval, the endogenous peroxidase activity was quenched by incubating the sections in 0.3% hydrogen peroxide for 30 min. Sections were blocked with Universal Blocking Buffer (Daco) for 10 min, following overnight incubation with specific antibodies against Calbindin antibody (1:200; Cell Signaling Technologies). Then tissues were incubated with biotinylated anti-rabbit IgG (Vector) for 1 h at room temperature, following incubation with Vestatin ABC solution. POD reaction was done by using DAB substrate (Vector). Counterstaining was performed with hematoxylin.

Images were digitally captured by a 12-megapixel Olympus DP71 camera for analysis. The cell density was analyzed by measuring the number of Purkinje cells per cell layer (linear density) using Metamorph software (Universal Imaging Corporation). For morphological analysis of Purkinje cells, the long axis of cell body was measured and taken as an indicator of cell size. All analysis were performed blind to the animals used. Results are reported as mean and S.E.M. Statistical significance was determined by Kolmogorov-Smivnov test and defined at P < 0.05.

### Corticosteroid measurement

Corticosteroid levels were measured in 10-week old KO (n = 19) and littermate WT (n = 12) control animals (both genders). Blood samples were taken in the morning (9.00 am) and evening (6.00 pm). Stress-induced corticosteroid levels were evaluated in blood samples from mice before and after physical restraint. Briefly, each mouse was individually placed in a plastic film decapicone (Braintree scientific inc., Braintree, MA, USA) for 30 min in a horizontal position. The animals were capable of breathing through the breathing hole at the smaller end and were immobilized without being squeezed. The larger end was tightly closed with a paper clip. All the subjects were checked to prevent suffocation. Serum concentrations of corticosteroids were measured using a commercially available Corticosterone 125I RIA kit (MP Biomedicals, USA), kindly provided by Dr. Daniel Drucker (SLRI).

### Tail flick test

Antinociceptive effects of the test substances were determined by the hot tail-flick method described by Sewell and Spencer [77]. One to two cm of the tail of a mouse was immersed in warm water kept at 50°C. The reaction time was the time taken by the mice to deflect their tails. The first reading is discarded and the reaction time was taken as a mean of the next two readings. The latent period of the tail-flick response was taken as the index of antinociception.

### Olfactory test

Mice were first habituated to the food (Bud's Best Cookies, Hoover, AL, USA) by the experimenter leaving food pellets (1 × 1 × 0.5 cm) in the home cage overnight. The next day, rodent food pellets were removed from the home cage, and the mice were food deprived for 24 hr. The test was conducted in a standard polycarbonate cage (30 × 17 × 12 cm). A piece of chow was placed in a randomly chosen area on the cage floor and then the entire cage floor was covered with corncob bedding to a depth of 2.5 cm. The subject was then placed into the cage and latency to find the food was recorded up to a maximum time limit of 15 min.

### Brain weight

For wet brain weight determination of KO and WT mice (n = 25 in each group) at 24 weeks old were sacrificed. The entire brain with olfactory bulbs and cerebellum were weighted.

### Statistical analysis

Data were analyzed by two-tailed *t *tests for independent samples; two- and three-way ANOVAs followed by Fisher least significance difference (LSD) post-hoc test. PPI was analyzed by two-way ANOVA with gene and gender as a between-subjects factors, and prepulse intensity as a repeated measurement factor. Latent Inhibition was by two-way ANOVAs with main factors of pre-exposure (0,40) and genotype (WT; GSK-3α KO). Values in figures are expressed as Mean ± S.E.M.

## Results

To address the effect of GSK-3α gene deficiency on behavior, we subjected GSK-3α KO mice and their wild-type littermates to a battery of well-established behavioral tests.

### Increased emotionality in GSK-3α female knockout mice

**Figure 1:**
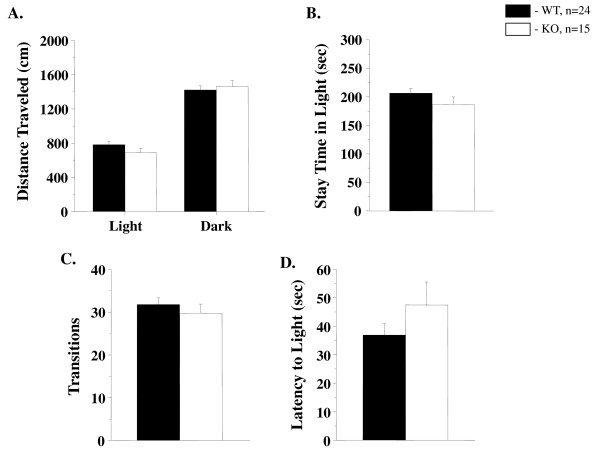
**GSK-3α male KO mice display no differences in light/dark transition tests**. In the light/dark transition test, GSK-3α KO (n = 15) and WT (n = 24) males were placed individually in the dark compartment at the start of the test. Data are given as mean (± SEM). **A**. Distance traveled (cm) in the light and dark sides. **B**. Time (sec) the mice stayed in the light side. **C**. Number of transitions between the light and dark sides. **D**. Latency time (sec) before the first entry into the light side. p.value ≥ 0.12 (n.s.)

The elevated plus maze (EPM) is a widely used test to measure anxiety-like behavior. The number of entries into the open arms and the time spent in the open arms are used as indices of open space-induced stress in mice [58]. GSK-3α KO females spent significantly more time in the closed arms and less in the open arms of EPM than WT littermates, suggesting increased stress behaviors in the KO females (Table 1). However, KO males in EPM behaved similarly to WT animals. Innate avoidance of the bright environment was measured by using an automated light/dark transition test. The number of transitions, distance traveled and latency to light in the light/dark transition test of GSK-3α KO males were all similar to WT littermate controls (Figure 1), confirming normal behavior of GSK-3α KO males in tests associated with increased stress.

**Table 1:** Analysis of anxiety-like behavior in GSK-3α female KO mice

Parameters (Number)	WT (n = 7) females	KO (n = 9) females	WT (n = 14) males	KO (n = 13) males
Passages (n)	1.57 ± 0.94	3.11 ± 1.56	0.42 ± 0.23	1.23 ± 0.52

Risk assessment (n)	3 ± 1.1	5 ± 1.21	4.57 ± 0.75	4.23 ± 0.58

Head dips (n)	10 ± 2.1	7.44 ± 1.16	9.71 ± 1.93	11.54 ± 1.7

Total number	58.14 ± 4.37	59.67 ± 3.2	54.21 ± 3.64	59 ± 4.64

Open (%)	16.56 ± 2.8	10.91 ± 2.67	12.39 ± 2.6	15.51 ± 2.16

Centre (%)	46.55 ± 1.34	45.18 ± 0.69	45.37 ± 0.98	44.31 ± 1.28

Closed (%)	36.89 ± 2.78	43.91 ± 2.62	42.24 ± 2.34	40.17 ± 2.14

**Parameters (Time)**	**WT (n = 7) females**	**KO (n = 9) females**	**WT (n = 14) males**	**KO (n = 13) males**

Open (%)	5.5 ± 1.18	2.62 ± 0.81 *	4.47 ± 1.04	5.7 ± 1.2

Centre (%)	25.11 ± 2.21	20.08 ± 1.68	21.39 ± 1.43	20.26 ± 1.72

Closed (%)	69.38 ± 3.14	77.3 ± 2.26 *	74.13 ± 1.78	74.04 ± 2.5

In the elevated plus maze, GSK-3α KO females (n = 9) showed significant increases in the percentage of the time spent in closed arms and decreases in the % of the time spent in the open arms compared with WT females (n = 7) in EPM. Values are mean (± SEM). *p ≤ 0.05.

To estimate emotional responsiveness in both groups, mice were subjected to a bright open-field arena (~200 lux) in a dim testing room for 5 minutes. GSK-3α KO animals of both genders showed a significant increase in freezing and grooming behavior (Table 2), indicating elevated instinctive behaviours in these animals compared with their littermate controls.

**Table 2:** Differential behavior of GSK-3α KO mice in the open field test

Parameters (Time, sec)	WT female (n = 7)	KO female (n = 8)	WT male (n = 13)	KO male (n = 13)
**Freezing**	2.64 ± 2.4	**19.07 **± 5.13 *	3.15 ± 1.18	**21.47 **± 4.94 * *
**Grooming**	3.87 ± 0.92	**13.4 **± 3.6 *	8.52 ± 2.68	**18.52 **± 2.68 * *
Rearing Wall	40.87 ± 4.62	33.31 ± 4.4	35.72 ± 4.14	34.02 ± 5.1
Rearing Open	5.16 ± 2.44	1.67 ± 0.58	4.97 ± 1.41	**1.32 **± 0.74 *
Activity Wall	253.21 ± 5.78	241.47 ± 5.7	162.09 ± 29.54	172.47 ± 31.45
Activity Centre	19.39 ± 3.86	12.91 ± 2.12	99.8 ± 28.01	75.76 ± 27.76
**Parameters (Number)**	**WT female (n = 7)**	**KO female (n = 8)**	**WT male (n = 13)**	**KO male (n = 13)**
Freezing	0.33 ± 0.33	6.33 ± 1.5 **	2.14 ± 0.78	8.23 ± 1.96 **
Grooming	2 ± 0.62	2.67 ± 0.75	2.79 ± 0.56	3.69 ± 0.41
Rearing Wall	34.71 ± 2.6	29.78 ± 2.56	29 ± 2.72	24.92 ± 2.4
Rearing Open	5.86 ± 2.8	2.22 ± 0.7	4.71 ± 1.42	1.08 ± 0.49 *
Activity Wall	55.86 ± 4.08	47.22 ± 2.59	34.79 ± 6.05	32 ± 5.42
Activity Centre	13.86 ± 2.54	7.89 ± 0.82 *	24.23 ± 4.75	17.15 ± 4.65

In the open field test, GSK-3α KO (n = 7-13) and WT (n = 8-13) mice were subjected to the bright open field arena in a dark room for 5 minutes. The duration and number of different parameters were measured (as indicated in the table). Values are mean (± SEM). *p ≤ 0.05; **p ≤ 0.001.

### Reduced depression-associated behaviors in GSK-3α knockout mice

**Figure 2:**
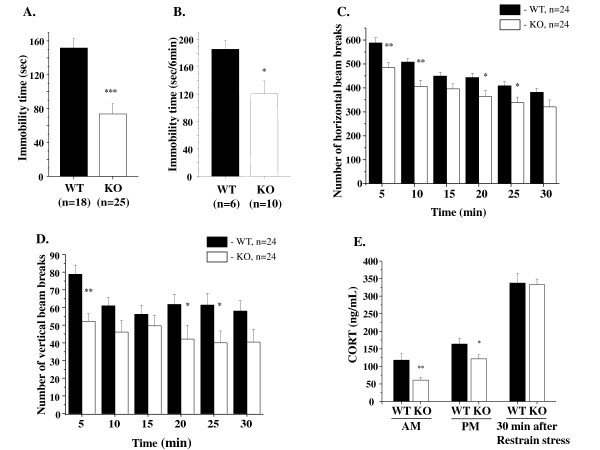
**Altered responses of GSK-3α KO mice in forced swim and tail suspension tests**. **A.** Forced swim test. Mean duration of immobility (± SEM) of GSK-3α KO (n = 25) and WT (n = 18) mice (both genders). Immobility time was decreased in KO group ***p ≤ 0.0001 vs. WT. **B**. Tail suspension test. Mean duration of immobility (± SEM) of GSK-3α KO (n = 10) and WT (n = 6) mice (both genders). Immobility time was decreased in KO group *p ≤ 0.05 vs. WT. **C-D.** Novelty-induced locomotion and exploratory activity in the open field. Mean number of beam breaks (± SEM) in 5 min bins by GSK-3α KO (n = 24) and WT (n = 24) mice (both genders). **C**. Horizontal locomotion was decreased in GSK-3α KO at following time points: 0-5, 5-10, 15-20, 20-25. **D**. Vertical (exploratory) activity was decreased in GSK-3α KO at following time points: 0-5, 15-20, 20-25. *p ≤ 0.05; **p ≤ 0.001. **E.** Basal and stress-induced corticosteroid levels. Basal morning (AM) and evening (PM) levels of corticosteroid (CORT) was measured at 9.00 am and 6.00 pm respectively in the naïve mice. Two weeks after, KO (n = 10-12) and WT (n = 19-20) animals were subjected to 30 minutes of restrain stress and blood samples were collected and analyzed for the level of CORT. Values are mean (± SEM). *p ≤ 0.05; **p ≤ 0.001

In the forced swim test (FST), rodents are placed in an inescapable cylinder partially filled with water, and the time spent immobile is measured over a short time window [78]; this test is a well-established paradigm for assessing motivational behaviors in rodents [79] and is used to predict antidepressant drug efficacy in both mice and rats [78]. Mice will generally swim in the water but after a while will stop swimming and float on the surface of the water, appearing to have given up trying to find solid ground. The duration of immobile floating on the surface of the water was decreased in GSK-3 mutants compared with WT littermates (Figure 2A). This finding was confirmed using a tail suspension test (TST) - a second paradigm commonly used to evaluate effects of antidepressants in rodents [65,79]. Mice are suspended by their tails for six minutes, during which time both activity and immobility are measured. Immobility time was significantly reduced for GSK-3α KO mice (Figure 2B).

Changes in general activity can affect animal behavior in FST and TST [79,80]. Locomotor activity in response to a novel environment was monitored in the open field. Compared to WT littermates, GSK-3α KO mice displayed reduced horizontal and vertical activity throughout the 30 minutes of the test period, indicating decreased locomotion and exploratory behavior in KO vs. WT animals (Figure 2C-D). Since the FST involves exposure to water, it increases stress in the animals as the animals prefer to avoid immersion. Stress stimulates the hypothalamus-pituitary-adrenal (HPA) axis, resulting in elevation of corticosteroid hormones. To determine whether the HPA response to stress was altered in GSK-3α KO mice, basal and stress-induced levels of corticosteroid (CORT) hormones were measured (Figure 2E). To check the status of basal levels of CORT in GSK-3α KO animals and their littermate controls, morning and evening measurements were taken. Basal CORT levels were significantly decreased in KO animals compared with WT in the morning as well as in the evening. After being subjected to restraint-induced stress, blood CORT concentrations increased and reached similar elevated levels in both groups (Figure 2E). These data indicate that GSK-3α KO mice have enhanced basal reactivity to stressful situations.

### Decreased social interaction and aggression in GSK-3α knockout mice

**Figure 3:**
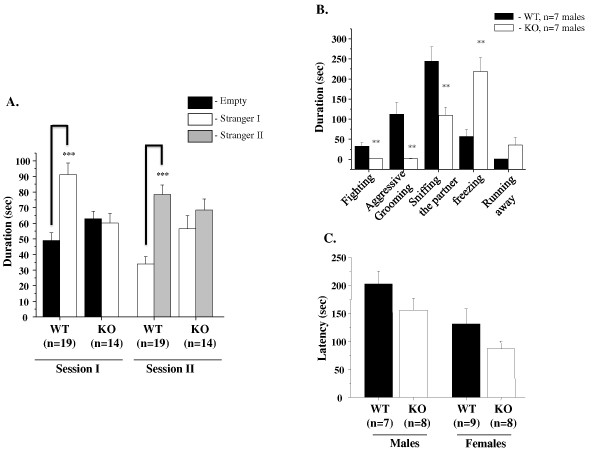
**Decreased social interaction and aggression in GSK-3α KO mice**. **A.** Social affiliation and social novelty. **Session I**. Measurement of sociability. Mean length of time (± SEM) in the chamber with the stranger ("stranger side") than in the opposite chamber ("empty side"). Unlike WT males (n = 19), KO (n = 14) animals failed to demonstrate a preference for social proximity by spending same time in both chambers. ***p ≤ 0.0001 in WT group. **Session II**. Measurement of social novelty. Mean duration of time (± SEM) in the chamber with the unfamiliar mouse from the sociability phase ("stranger 1") and in the opposite chamber with a new unfamiliar mouse ("stranger 2"). Unlike WT males (n = 19), KO (n = 14) animals failed to demonstrate a preference for social novelty by spending same time in both chambers. ***p ≤ 0.0001 in WT group. **B. **Resident Intruder. GSK-3α KO mice (n = 7) and their littermate controls (n = 7) were individually isolated for four weeks and after were tested in the presence of the "intruder" in the home cage. GSK-3α KO males showed a significant decrease in the duration of fighting and aggressive grooming. The KO group showed decreased social interaction including the time spent sniffing the partner compared with WT males. The time spent by KO males in freezing behaviors and running away from the opponent was increased vs. WT. Values are mean (± SEM). **p ≤ 0.001. **C.** Olfactory bulb test. First, mice were habituated to a new chocolate food in the home cage overnight. The next day, rodent food pellets were removed from the cage, and the mice were food deprived for 24 hours. KO (n = 8) or WT (n = 7-9) mice were placed in the new cage, and the latency to find the hidden food was recorded. Values are mean (± SEM). p.value = n.s.

Sociability and social novelty were estimated using two phases of social interaction tests (session I and session II). Unlike wild-type mice, GSK-3α mutants did not prefer the chamber containing an unfamiliar mouse (stranger 1) over an empty chamber in session I (Figure 3A). In addition, KO males did not favor the chamber containing a newly introduced mouse (stranger 2) over a chamber containing a now familiar mouse (stranger 1) in session II. Indifferent behavior of KO mice in this test is indicative of decreased social motivation and novelty (Figure 3A).

GSK-3α KO mice showed a significant decrease in duration of fighting and aggressive grooming and sniffing of the unknown intruder mouse compared with WT mice (Figure 3B). The time spent by GSK-3α KO males either freezing or running away from the opponent was increased vs. WT (Figure 3B). These findings indicate reduced aggression-like behavior in GSK-3α KO animals.

The lower social interaction and aggressive behavior of GSK-3α KO mice was not a result of diminished olfaction, as we found that the time required to find buried food in an olfactory test was not significantly different between the genotypes (Figure 3C).

### Altered information processing in GSK-3α knockout mice

Prepulse inhibition (PPI) and latent inhibition (LI) are the most common methods to quantify information processing in both humans and mice [81]. Sensorimotor gating is the process by which inhibitory neural pathways filter multiple stimuli and allow attention to be focused on one stimulus [82]. Sensorimotor gating can be measured by prepulse inhibition of startle, which is the modulation of the startle response by a weak prepulse [83]. The acoustic startle reflex is a simple neural circuit, involving approximately five synapses [84], and prepulse modification of this circuit has been postulated to arise from diverse cortical, midbrain, and hindbrain centers [85]. PPI is defined as the degree (%) to which the acoustic startle response is reduced when the startle-eliciting stimulus is preceded by a brief, low intensity, non-eliciting stimulus [86].

**Figure 4:**
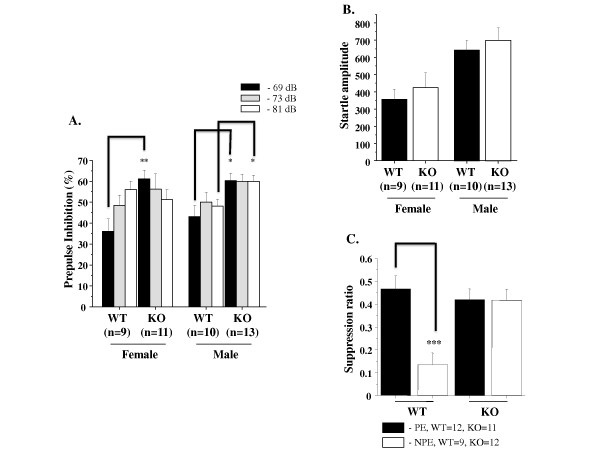
**Impaired information processing in GSK-3α KO mice**. **A-B. **Prepulse inhibition of acoustic startle response. **A**. PPI assay using a combination of startle (120 dB) and three prepulse levels (69 dB, 73 dB, and 81 dB) in GSK-3α KO (n = 11-13), WT (n = 9-10) (each gender). PPI is expressed as the mean percent reduction (± SEM) in startle amplitude at all three prepulses. Higher y axis values represent greater percent PPI. *p ≤ 0.05; **p ≤ 0.001. **B**. The amplitude of the acoustic startle response was similar between WT and KO. p.value = n.s. **C.** Latent inhibition. Mean suppression ratios (± SEM) of GSK-3α KO (n = 11-12) and WT (n = 9-12) (both genders). LI deficit was observed in GSK-3α KO group. PE = pre-exposed mice; NPE = non-pre-exposed mice to the CS. ***p ≤ 0.0001 in WT PE vs. NPE.

GSK-3α KO mice were tested for sensorimotor gating deficits in the prepulse inhibition test (Figure 4A-B). We found that GSK-3α KO mice have facilitated PPI at the lowest prepulse (69 dB) in KO females vs. WT and at 69 and 81 dB in KO males vs. WT (Figure 4A). The amplitude of the acoustic startle response was similar between KO and WT, however, a gender effect on ASR was observed (Figure 4B).

Latent inhibition refers to the phenomenon by which pre-exposure to stimuli (such as tones) retards subsequent conditioning to the same stimulus. The reduced conditioning is believed to result from attention filtering so that decreased attention is given to a familiar stimulus that the animal has learned to ignore during pre-exposure [87]. WT mice that were pre-exposed (PE) to the tone showed less freezing to the tone on the test day compared to non pre-exposed (NPE) animals (Figure 4C), indicating significant latent inhibition in control mice. The suppression ratio of pre-exposed GSK-3α KO mice was similar to that of non-exposed GSK-3α KO mice (Figure 4C), suggesting a deficit of the GSK-3α null mutant in latent inhibition.

### Long-term memory function in GSK-3α null mice

**Figure 5:**
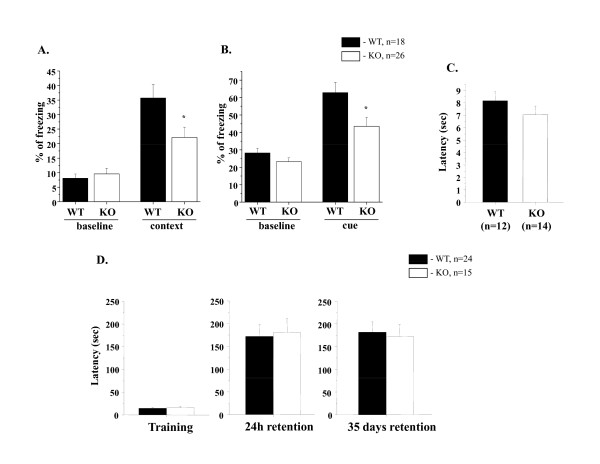
**Long-term memory function in GSK-3α KO mice**. **A-B. **Pavlovian fear conditioning (FC). Memory consolidation was assessed with a contextual and cued fear conditioning test. KO (n = 26) and WT (n = 18) mice were places in novel environment chamber for 3 min (conditioning stimulus, CS), where they received one electrical foot shock (unconditioning stimulus, US; shock intensity = 0.5 mV, duration = 2 sec). Twenty-four hours later mice were placed in the same (context) or modified (cue) chamber, and freezing time was measured as an indicator of memory consolidation. **A**. Contextual FC (baseline is a % of freezing before CS-US). **B**. Cued FC (baseline is a % of freezing before CS). KO mice (both genders) demonstrated less freezing in **A**. and **B**., indicating impaired contextual and cued FC. *p ≤ 0.05 in KO vs WT. **C.** Tail flick test. The index of antinociception was detected by hot tail-flick method in WT (n = 12) and KO (n = 14) mice. Values represent the mean time for tail flicking response following direct heat stimulation. No significant differences (p.v. = n.s) were found between the groups. **D.** Passive avoidance. GSK-3α KO (n = 14) and WT (n = 24) mice were tested for learned fear response to the shocked chamber. Mean latency (± SEM) scores entering into the dark chamber before electric shock; and 1 and 35 days after the electric shock are shown. No significantly differences were found between KO and WT groups (p.value = n.s).

Recently, GSK-3 has been reported to be involved in synaptic plasticity [24-27] and memory reconsolidation [53]. To examine whether the loss of GSK-3α was associated with cognitive deficits, we assessed memory and learning in GSK-3α KO mice in a Pavlovian fear conditioning paradigm as well as in a passive avoidance test (Figure 5).

GSK-3α KO mice showed similar freezing times in response to the unconditioning stimulus (US) compared to their littermate controls (baseline in Figure 5A and 5B). Memory performance in KO and WT groups was examined 24 hours after exposure to the US, by measuring freezing time (Figure 5). In the following trial (using the same chamber as used in the previous conditioning, without the sound), the learning freezing response of GSK-3α KO-context group was decreased (Figure 5A). In the cued trial (placing the mice into a different shape of chamber from that used for the conditioning, but with the presence of sound as was used for the conditioning), the GSK-3α KO-cue group showed a decreased level of freezing compare to WT, suggesting that GSK-3α mutant animals have an impaired ability to form and consolidate memory (Figure 5B). The decreased percent of freezing of GSK-3α KO mice in the FC test was not a result of diminished sensitivity, as we found that the latency in the tail flick test was not significantly different between the genotypes (Figure 5C).

However, no difference was found in the passive avoidance test (Figure 5D) between KO and WT groups; suggesting that the learning ability of KO was similar to WT and the behavior of the mutant group in the FC test might be interpreted as active avoidance of a fear-related situation leading to them freezing less than WT animals.

### Neurochemistry

To investigate whether altered levels of monoamines could contribute to some of the social, cognitive and memory deficits observed in GSK-3α KO mice, levels of catecholamines and their metabolites were measured using HPLC-electrochemical detection (Table 3). Slight but significant decreases were observed in the levels of HVA (in PFC) and NE (in hippocampus). However, the level of NM was increased in the amygdala (Table 3). Analysis of striatum revealed no significant alterations between genotypes in the profile of monoamines (Table 3).

**Table 3:** HPLC analysis of catecholamine neurotransmitters

	Dopaminergic		Norepinergic		Serotonergic	
	
Monoamine neurotransmitters (ng/mg of tissue)	DA	HVA	NE	NM	5-HT	5-HIAA
**Prefrontal Cortex**						

**WT (n = 9)**	0.041 ± 0.028	**0.102 ± 0.006**	0.48 ± 0.018	0.077 ± 0.003	0.426 ± 0.021	0.238 ± 0.01

**KO (n = 9)**	0.041 ± 0.018	0.086 ± 0.004*	0.47 ± 0.017	0.083 ± 0.006	0.394 ± 0.017	0.25 ± 0.007

**Hippocampus**						

**WT (n = 9)**	0.018 ± 0.001	0.038 ± 0.003	**0.651 ± 0.023**	0.056 ± 0.003	0.669 ± 0.019	0.705 ± 0.042

**KO (n = 9)**	0.015 ± 0.002	0.041 ± 0.003	0.566 ± 0.016**	0.051 ± 0.004	0.625 ± 0.016	0.747 ± 0.029

**Amygdala**						

**WT (n = 9)**	0.805 ± 0.041	0.303 ± 0.013	0.498 ± 0.018	**0.055 ± 0.003**	0.562 ± 0.021	0.466 ± 0.031

**KO (n = 9)**	0.835 ± 0.087	0.291 ± 0.012	0.506 ± 0.013	0.064 ± 0.003*	0.523 ± 0.021	0.492 ± 0.021

**Striatum**						

**WT (n = 9)**	13.49 ± 0.42	1.486 ± 0.049	0.12 ± 0.009	0.029 ± 0.003	0.425 ± 0.023	0.407 ± 0.028

**KO (n = 9)**	13.486 ± 0.28	1.534 ± 0.036	0.117 ± 0.006	0.024 ± 0.002	0.42 ± 0.017	0.399 ± 0.01

Levels of monoamines and metabolites, including dopamine (DA), 4-hydroxy-3- methoxyphenylacetic acid (HVA), 3 norepinephrine (NE), normetanephrine (NM), serotonin (5-HT) and 5-hydroxyindoleacetic acid (5-HIAA), were quantified by HPLC with electrochemical detection in prefrontal cortex (PFC), hippocampus (HIP), amygdala (AM) and striatum (STR) of GSK-3α KO (n = 9) and WT (n = 9) mice. In bold type *p.value ≤ 0.05; **p.value ≤ 0.01 KO vs. WT.

### Neuroanatomical and histological examination of the brain

**Figure 6:**
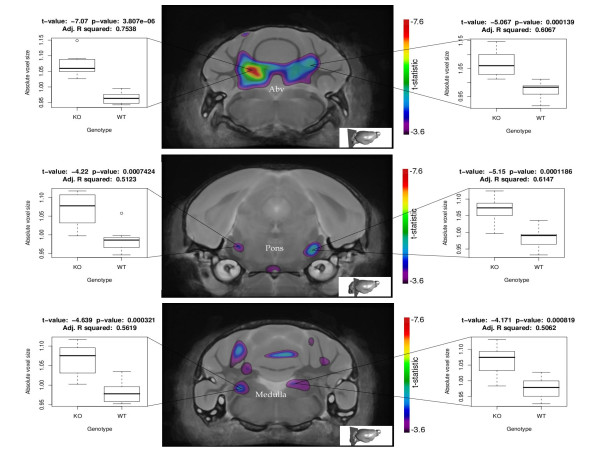
**MRI of the brain**. The per-voxel results of the statistical map of the Jacobian determinant (tissue compression/expansion) are shown as composite images of two coronal sections of the GSK-3α KO brain compared to WT control brain. All colored voxels are significant with a false discovery rate of 10% (n = 9 for knock outs, n = 8 for wild types). The coronal level of each section is pictorially illustrated in the bottom right corner. Abv, arbor vita of the cerebellum, CbCx, cellebellar cortex, CrAq, cerebral aqueduct.

We examined 9-10 week old GSK-3α mice for structural brain abnormalities using very high resolution MRI combined with computer modeling (Figure 6). Analysis by MRI revealed no overall brain size difference. We used a statistical map of the Jacobian determinant that illustrates the expansion and contraction of tissue based on genotype, to find regions of significant change. In order to account for an inflated amount of false positive findings due to the number of statistical tests employed, the FDR technique was applied with a 10% FDR threshold. The arbor vita of the cerebellum of the GSK-3α knockout mice was observed to be significantly enlarged by 9% compared to wild type mice (Figure 6). The mean volume was 12.8 ± 0.55 mm^3 ^in the knock out animals and 11.7 ± 0.4 mm^3 ^in wild types. The pons was found to be significantly larger by 5% in the knock out animals. Mean volume was 18.1 ± 0.67 mm^3 ^in the knock outs and 17.2 ± 0.4 mm^3 ^in wild type samples (Figure 6). Slightly increased volume of medulla oblongata was observed in KO (24.48 mm^3^) vs WT (23.65 mm^3^) mice (Figure 6). Small regions of significantly expanded voxels were also found in the cerebellar cortex, the cerebral aquaduct, the cerebral cortex, the cerebellar peduncle, the left striatum and the right olfactory bulb, as well as a small region of significantly contracted voxels in the parieto-temporal lobe of the cerebral cortex. No gender related differences were found.

**Figure 7:**
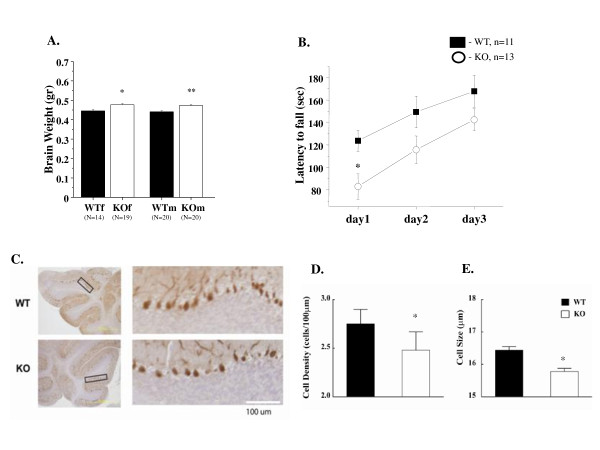
**Neuroanatomical and histological examination of the brain**. **A. **Brain weight. Brain weight analysis was perform in 24 weeks GSK-3α KO (n = 19-10) and WT (n = 14-10) animals. Values are mean (± SEM). *p ≤ 0.05; **p ≤ 0.001. **B. **Rotarod. GSK-3α KO (n = 13) and WT (n = 11) mice were tested on an accelerating rotarod in 3-day trials. Significant differences between the groups (p.value = 0.013) were found on day1 in the balance and coordination; however no changes were found in motor learning. **C-D.** Decrease in Purkinje cell density and size in GSK-3α KO mice. **C**. Purkinje cells image. Immunohistochemistry against Calbindin antibody of WT and GSK-3α KO cerebellum (shown original magnification, ×4 (upper panel) and ×10 (lower panel)). **D**. Statistical analysis of Purkinje cell density. Average cell density was determined by taking the ratio of the number of Purkinje cells to the length of cerebellar cortex segments. Cell density is significantly decreased in the GSK-3α KO (p < 0.05; Kolmogorov-Smirnov Z Test). **E**. Significant decrease of cell size in the GSK-3α KO (p < 0.01; Kolmogorov-Smirnov Z Test). Cell size was determined by measuring the longest axis of Purkinje cell bodies [N = 2345 WT, N = 1588 GSK-3α KO (number of cells)].

Brain weight analysis of 6 months old animals revealed slightly but significantly increased brain weight of KO males and females compared with their littermate controls (Figure 7A). Thus, structural changes (especially in the cerebellum) found by MRI may correlate with increased brain weight of KO animals.

The cerebellum is an important component of the brain motor system, involved in the control and adaptability of movement. To elucidate the involvement of the cerebellum in morphological changes in GSK-3α null mice, we performed a cerebellum-dependent behavior test (Figure 7B). Associative motor learning, coordination and balance were examined by a rotarod test in KO and WT animals. A significant coordination deficit was observed in the KO group, as assessed by decreased latency to fall, compare to WT littermates. During three day trials, relative improvements in rotarod performance were found to be comparable between GSK-3α KO and WT mice (Figure 7B).

The cerebellar cortex contains eight types of neurons: Purkinje, granule, stellate, basket, Golgi, Lugaro, unipolar brush cells and candelabrum cells [88-90]. Purkinje cells provide the sole output of the cerebellar cortex and are the pivotal element around which the cerebellar circuit is organized [88]. Most Purkinje cells express calbindin, which is expressed when neurons start to migrate and differentiate [91]. By using anti-calbindin immunohistochemistry, we examined the Purkinje cell composition of the cerebellum in 9-10 week old KO and WT animals (Figure 7C). We quantified cell density and shape, analyzing more than 1500 calbindin-positive cells per sample. Significant decreases in Purkinje cell density and size were observed in GSK-3α KO mice (Figure 7D-E).

## Discussion

We recently generated mice that lack GSK-3α. These animals are viable and show increased insulin sensitivity and liver glycogen accumulation [57]. We have performed comprehensive analyses of brain functions of these mice, including extensive behavioral examination, evaluation of neurotransmitter levels and neuroanatomical studies. Similar to GSK-3β^+/- ^mice [29,50,52], GSK-3α mutants have decreased exploratory activity, decreased immobility time and anti-aggression behavior. To our knowledge, this is the first *in vivo *study showing several abnormal behavioral features unique to mice lacking the GSK-3α genes, such as decreased locomotion, increased sensitivity to environmental cues, decreased social motivation and novelty; impaired sensorimotor gating, associative memory and coordination.

Several genetic approaches have been used to generate mutant mice for GSK-3, including conventional GSK-3β^+/- ^[48,53] and GSK-3α^-/- ^mice [57], conditional Nestin-specific GSK-3α, β knockout [55] and Nestin-GSK-3α, β knockdown mice [92], GSK-α, β knock-in [56,93] as well as mice overexpressing GSK-3β [94]. Studies in GSK-3β^+/- ^mice have shown increased anxiety-associated behavior [51], which is similar to our findings in GSK-3α KO females. However, GSK-3α KO males have normal performance in EPM and light/dark transition test, indicating comparable behavior to their WT littermates. In addition, a significant increase in grooming activity was observed in GSK-3α KO mice (both genders). A summary of the behavioral responses of the GSK-3α KO animals compared to wild type littermates is shown in Table 4.

**Table 4:** Summary table of behavioral phenotypes of GSK-3α KO mice vs. WT littermate controls

Type of behavior	Changes	Type of the test	Figure #
**Anxiety**	▲ in females only	EPM	Table 1
	
	**≠ **in males	Light-dark box	Figure 1A-D

**Emotionality**	▲ (both genders)	Open field 5 min	Table 2

**Locomotor activity**	▼	Open field 30 min	Figure 2C

**Exploratory activity**	▼	Open field 30 min	Figure 2D

**Depression-like behavior**	▼	FST	Figure 2A
	
	▼	TST	Figure 2B

**Sociability and social novelty**	▼	Social interaction test	Figure 3A

**Aggression**	▼	Resident Intruder	Figure 3B

**Information processing**	▲ PPI	PPI/ASR	Figure 4A-B
	
	▲ NPE in KO	LI	Figure 4C

**Long term memory**	▼	FC	Figure 5A-B
	
	**≠**	Passive avoidance	Figure 5D

**Coordination, balance**	▼	Rotarod	Figure 7B

**Motor learning**	**≠**	Rotarod	Figure 7B

**Neuroanatomical changes**	▲ cerebellum	MRI	Figure 6

	▲ brain	Brain weight	Figure 7A
	
	▼ Purkinje cells	IHC	Figure 7C-E

▲ (black block upward-facing arrow) - increased; ▼ (black block downward-facing arrow) - decreased; **≠ **(crossed out equal sign) - same/no changes

GSK-3α KO mice exhibited decreased immobility times in FST and TST. Increased floating or mobility in these animals was not due to hyperactivity. In contrast, GSK-3α null animals demonstrated reduced locomotor activity in the open field test, which was not accompanied by changes in the level of dopamine in the striatum. A similar behavior in the FST was observed in mice harboring a deletion of one allele of GSK-3β [29,50], but it is interesting to note that locomotion in these animals (GSK-3β^+/-^) was unchanged [29,50]. Reduced immobility time has been also shown in mice overexpressing GSK-3β, but that phenotype was related to hyperactivity of those animals, as observed in an open field test [54]. The effect of the GSK-3 inhibitor, lithium [39,40], to decrease immobility time in FST, was first reported in rats [95] and was later replicated in mice [96].

Increased mobility time during FST and TST, has been interpreted as active, attempted avoidance of a stressful situation. Examination of basal corticosteroids revealed significantly decreased levels in KO mice, but stress-induced levels were similar between the groups. On the other hand, no changes in basal and stress-induced corticosteroid levels were detected in mice overexpressing GSK-3β [54]. Corticosteroid receptors in the brain - glucocorticoid receptors (GR) and mineralocorticoid receptors (MR) play an important role in modulating the HPA [97] and their altered function has been implicated in the pathogenesis of psychiatric disorders [97]. FbGRKO (forebrain-specific GR KO) mice have hyperactivation of the HPA axis, impaired negative feedback regulation of the HPA axis and, increased depression-like behavior [98]. On the other hand, increased GR and/or MR activity in the hippocampus normalizes the hyperactivity of the HPA axis, because the hippocampus plays a major role in mediating negative feedback of glucocorticoids [97]. GSK-3 has been shown to phosphorylate and inhibit GR [99]. Moreover, chronic treatment with a GSK-3 inhibitor, lithium, increases the mRNA level of GR in the hippocampus and hypothalamus [100]. It is possible that inactivation of GSK-3α may affect the function of HPA axis and/or GR and thus may contribute to the abnormal sensitivity to stressful situations in the GSK-3α KO mice.

We observed changes associated with social interactions in GSK-3α null males when compared to control animals. We found robust decreased social interaction in the KO animals in both sessions of this test. When assessed within the resident-intruder paradigm to reveal aggressive behavior, significantly fewer attacks were made towards the intruder and GSK-3α KO males tended to move away from the opponent. These observations indicate significant disruption in territorial aggression in the GSK-3α knockout animals, an important component of social behavior in adult males. Similar to GSK-3α KO mice, anti-aggression behavior was observed in GSK-3β^+/- ^mice [52]; however, parameters of sociability have yet to be assessed in GSK-3β^+/-^mice.

The mechanism underlying decreased social behavior in GSK-3α KO mice is still unknown. It is known that social behavior is affected by an animals sentiment (e.g. familiarity and comfort within a given situation) [101], hence, the increased sensitivity to stress observed in GSK-3α KO mice might explain their decreased social affiliation/novelty and defensive behavior during the direct social contacts with intruder. Moreover, of the distinct behaviors results may be explained in terms of stress responsiveness of the GSK-3α KO mice. Each test is associated with a level of aversive conditions and, hence, exposure of animals to a new situation in the behavioral test will naturally trigger stress. Importantly, the GSK-3α KO mice expressed very mild changes in the EPM test which is relatively non-stressful experience. However, the bright, inescapable open field test, as well as the unfamiliar partner in the resident-intruder task, stimulated passive avoidance in KO animals. Finally, the highly aversive situations in the FST or TST provoked greater active avoidance behavior in GSK-3α KO mice.

Norepinephrine (NE) is a stress hormone, and we note that small but significant reductions in NE levels were observed in the hippocampus of GSKα KO animals. Mouse models engineered to lack NE [102,103] display anxiety, impaired social memory and low levels of aggression [104], as well as reduced exploratory activity and decreased locomotion in novel environment, open field situations [105], phenotypes similar to those we observe in GSKα KO animals. Thus, the decreased level of NE in the hippocampus of GSKα^-/- ^KO mice may contribute to reduced sociability and aggressive behavior in these animals. Further studies will elucidate this question.

Previous studies have reported that GSK-3β protein levels in the cortex of different inbred mouse strains are inversely correlated with strength of prepulse inhibition (PPI) [106]. The prepulse inhibition enhancement observed in the GSK-3α KO mice suggests that GSK-3α may contribute to normal sensorimotor gating. The limbic and cortico-pallido-striato-thalamic circuitry is thought to be responsible for modulation of PPI in the rodents. Thus, these results may reflect dysfunction within the auditory-startle response pathways, which consists of an essential central relay in the cochlear nuclear complex, an intermediate brain stem relay in the reticular formation, a long reticulospinal pathway via the medial longitudinal fasciculus and outputs via spinal cord and brain stem motoneurons [107]. Slightly increased volumes of the medulla oblongata and pons in GSK-3α KO mice were identified by MRI, indicating that anatomical changes in brain stem structure may contribute to alterations in sensory-motor-gating in the mutant animals. It is unlikely that the observed difference in PPI between the GSK-3α KO and WT groups was the result of poor hearing in the mutants. The magnitude of ASR (acoustic startle response) was similar between KO and WT groups of both genders. Results from pharmacological analysis [108-110] and mouse inbred strains [111] have clearly dissociated the startle and prepulse inhibition responses. In contrast, GSK-3β heterozygous mice and wild type mice treated with lithium show no changes in PPI and ASR [50,51]. Changes in psychomotor function and an increased acoustic startle response have been found in mice overexpressing GSK-3β [54,94].

Long-term potentiation (LTP) and long-term depression (LTD) are two forms of synaptic plasticity and have been implicated in the molecular and cellular basis of learning and memory [112,113]. Several recent studies report the importance of GSK-3 activity during synaptic plasticity and memory function. Activation of GSK-3 inhibits the development of long-term potentiation (LTP) [27], whereas its inhibition prevents the development of long-term depression (LTD), as shown in brain slice studies [24-26]. Transgenic mice overexpressing GSK-3β have reduced LTP induction [28] and impaired acquisition of reference memory in a novel object recognition task [114]. In addition, it has been shown that overexpression of GSK-3β impairs spatial learning [115], though the mechanism underlying this effect in unknown. Recent studies by Kimura *et al*. [53] have shown the importance of GSK-3β in memory reconsolidation in adult brain. Mice heterozygous for GSK-3β exhibit retrograde amnesia [53]. These animals have reduced memory reconsolidation but normal memory acquisition, suggesting that they might be impaired in their ability to form long-term memories.

A potential role for GSK-3α in cognitive function was evaluated by classical contextual and cued fear conditioning as well as by a passive avoidance test. The fear conditioning test can be used to examine both hippocampus-dependent memory and amygdala-dependent emotional memory [116-118]. GSK-3α KO mice exhibited impaired contextual and cued memory in the fear conditioning paradigm; however memory performance was comparable to WT in the passive avoidance test. Thus, it is possible that GSK-3α KO mice have relatively normal memory formation, but show active avoidance from stressful situations (in FC, FST, TST). Notably, the formation of associated memory was impaired in GSK-3α KO compared to WT mice as was observed in the latent inhibition test. This type of memory formation (LI) has not been tested in GSK-3β heterozygotes. Long-term treatment with a GSK-3 inhibitor, lithium, has been shown to impair latent inhibition and acquisition of conditioned response in rats which had not been pre-exposed to the stimulus [119]. It has also been reported that while lithium appeared to decrease the passive avoidance response to shock, its effect in an active avoidance paradigm was unchanged, leading to the suggestion that lithium decreases not cognition, but sensitivity to low-intensity stimuli [120,121]. It is possible that different neuronal circuits are affected by inactivation of GSK-3α which are differently involved in fear conditioning, passive avoidance and/or latent inhibition and memory formation. However, molecular and electrophysiological mechanisms underlying the effects of GSK-3α on learning/memory have yet to be elucidated.

Our studies revealed an increase in cerebellar volume, but normal brain volume in GSK-3α KO mice. In contrast, mice overexpressing GSK-3β have been reported to exhibit reduced brain volume, as determined by MRI, findings that have been ascribed to a reduced size in the somatodendritic compartments [94]. The cerebellum is an important brain region responsible for motor coordination [122,123], sensory [124] and cognitive function [125-128] and has also been implicated in emotional processing [129-131]. We examined cerebellum-dependent coordination and memory using a rotarod test. In contrast to GSK-3β heterozygote mice [50,51], GSK-3α KO mice showed impaired coordination in the rotarod test; however, motor learning in this test was unaffected.

Purkinje cells are considered to be the primary site of informational processing in the cerebellum and they channel output to the deep cerebellar and brainstem nuclei [132]. Immunohistological examination of the cerebellum of GSK-3α KO and WT animals revealed a significantly reduced number and size of Purkinje cells in the KO animal group. Considering these data together with neuroanatomical changes of cerebellum, we hypothesize that the expenditure of the region, observed by MRI, may correlate with morphological changes in deep cerebellar nuclei (DCN). Axons of PC are terminated in DCN [132]. A decreased number and size of PC, and possiblly their axons, may trigger compensatory changes in the density of DCN's dendrites and therefore may explain the increased volume in this region. Further experiments will elucidate the role of GSK-3α in cerebellar development and formation of cerebellar circuits.

Our findings raise the question of how loss of GSK-3α function may lead to the observed cerebellar phenotype? GSK-3 is involved in Reelin, Wnt, Notch and Hedgehog signaling pathways [5,7,8,133], which have been shown to be critical for cerebellar development. Early patterning of the cerebellum is mostly regulated by Wnt 1 [134,135] and the transcriptional factor Engrailed (En 2) [136-138]. These gene products are essential for cerebellar development and foliation. En2 has been shown to regulate genes related to vesicle formation and transport in Purkinje cells [139]. Notch signaling has been shown to regulate the differentiation of cerebellar progenitors through the period of cerebellar neurogenesis [140]. Shh (Hedgehog) signaling is critical for granule cell proliferation [141] and for the cerebellar foliation process [142-144]. The Reelin-to-Dab1 pathway [145-147], mediated by cadrerins [148-150] and integrins [151] has been implicated in the development of Purkinje cell-cell adhesion properties [152]. Decreased levels of Reelin [145,153] and the pro-survival molecule Bcl-2 have been suggested to play a role in the cerebellar pathology [153,154]. Lastly, lithium has been speculated to synergize with cytokines and neuroleptics and thereby disrupt calcium homeostasis within Purkinje cells [155]. Lithium toxicity is most prominent in the cerebellar cortex [156]. In humans, lithium toxicity is associated with severe loss of Purkinje cells [157-160]. Thus, GSK-3α may be required for normal Purkinje cell development and maintenance.

## Conclusion

We have identified GSK-3α as a factor influencing specific stress-response and social behaviors, as well as cerebellar physiology. The GSK-3α mutant mice provide a new genetic model to study factors that may influence abnormalities in these processes.

## Competing interests

The authors declare that they have no competing interests.

## Authors' contributions

JRW is responsible for the original concept and overall design of the research. OKB, TVL, KT, SH, TM, JR performed behavioral analysis of mutant mice. OKB, MK, KO performed the immunohistochemistry study and analysis of the data. OKB, MVE, CL, MRH performed MRI experiments. OKB, JC, PF performed HPLC analysis. OKB performed corticosteroid experiments. OKB, KM, BWD performed GSK-3α KO animal characterization. OKB and JRW wrote the manuscript. All authors have read and approved the final manuscript.
